# Prospective evaluation of vesicourethral anastomosis outcomes in robotic radical prostatectomy during early experience in a university hospital

**DOI:** 10.1590/S1677-5538.IBJU.2016.0466

**Published:** 2017

**Authors:** Lucas Medeiros Burttet, Gabrielle Aguiar Varaschin, Andre Kives Berger, Leandro Totti Cavazzola, Milton Berger, Brasil Silva

**Affiliations:** 1 Departamento de Urologia, Hospital de Clínicas de Porto Alegre, RS, Brasil; 2 Universidade Federal do Rio Grande do Sul, RS, Brasil; 3 Institute of Urology, University of Southern California, Los Angeles, CA, USA; 4 Departamento de Cirurgia Geral, Hospital de Clínicas de Porto Alegre, RS, Brasil

**Keywords:** Minimally Invasive Surgical Procedures, Prostatectomy, Urinary Incontinence

## Abstract

**Purpose:**

Robotic assisted radical prostatectomy (RARP) presents challenges for the surgeon, especially during the initial learning curve. We aimed to evaluate early and mid-term functional outcomes and complications related to vesicourethral anastomosis (VUA), in patients who underwent RARP, during the initial experience in an academic hospital. We also assessed possible predictors of postoperative incontinence and compared these results with the literature.

**Materials and Methods:**

We prospectively collected data from consecutive patients that underwent RARP. Patients with at least 6 months of follow-up were included in the analysis for the following outcomes: time to complete VUA, continence and complications related to anastomosis. Nerve-sparing status, age, BMI, EBL, pathological tumor staging, and prostate size were evaluated as possible factors predicting early and midterm continence. Results were compared with current literature.

**Results:**

Data from 60 patients was assessed. Mean time to complete VUA was 34 minutes, and console time was 247 minutes. Continence in 6 months was 90%. Incidence of urinary leakage was 3.3%, no patients developed bladder neck contracture or postoperative urinary retention. On multivariate analysis, age and pathological staging was associated to 3-month continence status.

**Conclusion:**

Our data show that, during early experience with RARP in a public university hospital, it is possible to achieve good results regarding continence and other outcomes related to VUA. We also found that age and pathological staging was associated to early continence status.

## INTRODUCTION

Radical prostatectomy (RP) is the standard surgical therapy for localized prostate cancer, which is the second most common solid neoplasm in men worldwide and the fourth cause of cancer death (1). In Brazil, it occupies the first position regarding incidence of cancer in men, and it is the second cause of mortality (2).

Robotic assisted radical prostatectomy (RARP) has become the most commonly performed surgical technique in several countries, and is increasingly being employed in Brazil (3). Advocated advantages of robotic surgery are decreased blood loss and other complications, better early continence and sexual function, less positive surgical margins, and also diminished hospital stay and early return to regular activities (4–6).

Some technical features of the robotic system as the three-dimensional image with magnification, wristed instrumentation, and prevention of biological tremor may help on performing the challenging steps of RARP like bladder neck and neurovascular bundle dissection and vesicourethral anastomosis (VUA). Among those, VUA is one of the most technically demanding since it requires a watertight, tension-free suture, and minimal tissue damage, in order to obtain adequate healing. Even when performed with robotic assistance, an inadequate anastomosis may result in major complications like urinary leakage, prolonged urethral catheterization, increased length of stay, incontinence and bladder neck sclerosis (7). Overall complication rates with RARP may reach approximately 1.5% to 17.8%, even after the learning curve (8).

Although it is already widespread in the United States and Europe, RARP is still under implementation in several centers in Brazil and other countries, therefore we believed that it is crucial to evaluate results and complications during our early experience.

Considering the above mentioned, and since there is still few published data originated from robotic programs in our country, our objective was to assess early and mid-term functional outcomes and complications related to VUA, and to evaluate possible predictors of continence, in patients who underwent RARP during the initial experience in an academic hospital. We also compared these results with the literature.

## MATERIALS AND METHODS

We conducted a prospective study from August 2013 to August 2015 in the urology department at Hospital de Clínicas de Porto Alegre (Porto Alegre, RS, Brazil).

Patients with clinically localized prostate cancer who were candidate to primary treatment were offered RARP. Data was collected prospectively from consecutive patients, and those with at least 6 months of follow-up were included in the study. Operations were performed by two surgeons using the da Vinci SI robotic system with dual console. A total of 63 patients were submitted to RARP and VUA using monofilament barbed suture. The first 24 cases were mentored by an expert robotic surgeon.

### Surgical Technique

Radical prostatectomy was performed transperitoneally in the following sequence: dissection of Retzius space, dorsal venous complex ligation, bladder neck incision, vas deferens ligation, seminal vesicles dissection, lateral prostatic pedicles and antegrade nerve bundle dissection, apical dissection and pelvic lymph node dissection (PLND), when indicated. We use a four-arm robotic approach for port placement, with the third working robotic arm positioned on the right and one assistant port on the left flank, as described by Chopra and colleagues (9). After completion of RP and PLND, posterior reconstruction with a modified Rocco Stitch (RS) and VUA were performed according to Van Velthoven's technique (10, 11). A 18F Foley catheter was left usually for 7 days.

RS and VUA were performed using V-LOC™ 90 3-0 CV-23 (17mm needle) or V-20 (26mm needle), depending on surgeon's preference. At the end of surgery a 15F Blake drain was placed in the pelvis through the right (3rd arm) robotic trocar.

#### Outcome Measures

Data was collected prospectively using standardized institutional protocol. Preoperative, demographic and postoperative data were recorded.

Surgical outcomes were time to perform both the RS and VUA individually. Also, intraoperative events related to suture material were recorded (suture breakage, shearing of urethra or bladder neck, loss of tension). Operative times were recorded by stopwatch during video playback of all cases. The VUA time was measured from the first bite on the bladder until confirmation of a watertight VUA, by filling of the bladder with 120mL of saline. Estimated blood loss (EBL) and transfusion rates were also recorded, since bleeding during or after surgery has been suggested to negatively affect the quality of anastomosis and to be a predictor of urinary leakage (12).

Early postoperative complications were defined. Urinary leakage was considered as persistent drainage (more than 2 days) from drain or surgical incision, confirmed by elevation of creatinine in the fluid, or by contrast cystography. Cystography was not routinely used. Incidence of ileus was also presented and it was defined as requiring nasogastric tube placement as a result of an inability to resume a normal diet.

During follow-up, mid-term outcomes were evaluated prospectively during clinic visits. Urinary retention requiring catheterization, bladder neck sclerosis and continence were recorded.

Continence was assessed by phone calls or during visits to clinic on months 1, 3 and 6, by asking patients for need and number of pads used per day.

Possible predictors of continence were evaluated. Nerve-sparing status, age, BMI, EBL, pathological tumor staging, and prostate size were included as possible influencing factors and were compared to continence at 1, 3 and 6 months. Prostate size was derived from TRUS or MRI studies. Clinical and pathological stages were reported according to the 2009 TNM system and subcategorized into two groups: localized (pT2) or locally advanced disease (pT3 and pT4).

Nerve-sparing status was recorded prospectively according to surgeon's subjective assessment after surgery, or during video playback. Grading was defined for each neurovascular bundle as: non-nerve sparing, partial nerve-sparing or total nerve-sparing. For statistical analysis patients were subcategorized into two groups of preservation: at least 1 bundle totally spared or neither bundle spared.

Baseline characteristics and outcomes of all patients are presented as median (interquartile range) or mean (standard deviation) for continuous variables and frequencies and percentages for categorical variables. Logistic regression was used for univariate and multivariate analyses. A level of statistical significance of p<0.25 was considered for including variables on a final multivariate model for predictors of incontinence. Reported p values were 2-sided and statistical significance was set at p<0.05 on multivariate logistic regression. All statistical tests were performed using SPSSv.18 (IBM Corp, Armonk, NY).

The procedure with its benefits and all possible complications was explained to the patients and all participants signed a written consent. The study was approved by the Local and National Ethics and Research Committee.

## RESULTS

A total of 63 patients were consecutively submitted to RARP during the described period. Three patients were excluded from the analysis, two because they were operated by visiting surgeons from other institutions, and one because VUA was performed using monofilament non-barbed suture. Two patients had previous transurethral resection of the prostate. Sixty patients were included in the final analysis.

Median patient age was 64 (interquartile range 62–70). Median BMI value was 26.2 (23.8–29.2). Median baseline PSA value was 6.3ng/mL (5–8.2). The clinical stage of the primary tumor was cT1c in 28 (47%) patients, cT2a in 13 (22%) patients, cT2b in 4 (7%) patients, cT2c in 14 (23%) patients, and cT3 in 1 (2%) patient. Preoperative demographics of patients are summarized in Table-1. Table-2 shows postoperative pathological tumor staging.

**Table 1:** Patients’ demographics.

		n = 60
Age, years		64 (62-70)
BMI		26.2 (23.8-29.2)
Preoperative PSA, ng/mL		6.3 (5-8.2)
**Clinical T stage (n, %)**	cT1c	28 (47%)
	cT2a	13 (22%)
	cT2b	4 (7%)
	cT2c	14 (23%)
	cT3a/b	1 (2%)
**Gleason score Biopsy (n, %)**	6	27 (45%)
	7 (3+4)	21 (35%)
	7 (4+3)	7 (11.7%)
	8	4 (6.7%)
	9	1 (1.7%)
**Prostate size at TRUS (median; range)**		35.5 (28.1 – 46.1)

**median** (interquartile range); **TRUS:** transrectal ultrasound

**Table 2:** postoperative pathological staging.

	n=60	
Pathological Staging; n (%)	pT2a	10 (16.7%)
	pT2b	7 (11.7%)
	pT2c	33 (55%)
	pT3a	4 (6.7%)
	pT3b	5 (8.3%)
	pT4	1 (1.7%)

The perioperative outcomes are summarized in Table-3. Mean time to complete VUA was 34 minutes, and to complete RS median time was 8 minutes. The mean total procedure duration and console time was 298, and 247 minutes respectively. Mean EBL was 95mL (±158). No patients had blood transfusions during surgery, but two had postoperative bleeding requiring transfusion. Patients maintained Foley catheter for a median period of 7 days.

**Table 3:** perioperative outcomes.

Variable	n = 60
Total procedure time (min)	298±73
Console time (min)	247±65
Rocco Stitch time (min)	8±6
VUA time (min)	34±16
Estimated blood loss (mL)	95.7±158
Transfusions (n, %)	2 (3.3%)
Length of stay (days)	3 (2–5)
Catheter duration (days)	7 (7–10)
J-Blake drain duration (days)	3 (2–3)

Mean±standard deviation; Median (interquartile range)

There were no relevant intraoperative events regarding suture material adequacy. We did not observe shearing of tissues, back slippage or suture breakage.

Regarding early and mid-term complications, two patients (3.3%) were diagnosed with urinary leakage, both of them treated conservatively without need for surgical reintervention. Ileus requiring nasogastric drainage occurred in 3 (5%) patients, and these were not the same patients that presented urinary leakage (Table-4). With an average follow-up of 18.6 (±8.2) months, no patients have developed bladder neck contracture.

**Table 4:** Complications related to VUA.

Complications	n (%)
Urinary Leakage	2 (3.3%)
Ileus	3 (5%)
Urinary Retention	0
Bladder Neck Contracture	0

Table-5 shows our continence results. Continence (defined as no pad use or only one safety pad) was 62.1%, 76.7% and 90% in 30 days, 3 months and 6 months respectively. When considering continent patients only those that reported using zero pads, our 6-month result was 78.3%.

**Table 5:** Funcional outcome.

Urinary continence over time (pads/day)		N, N {missing}
**1 month**		**58 {2}**
	0	26 (44.8%)
	1	10 (17.2%)
	**0-1**	**36 (62.1%)**
	2	10 (17.2%)
	>2	12 (20.7%)
**3 months**		**60 {0}**
	0	39 (65%)
	1	7 (11.7%0
	**0-1**	**46 (76.7%)**
	2	8 (13.3%)
	>2	6 (10%)
**6 months**		**60 {0}**
	0	47 (78.3%)
	1	7 (11.7%)
	**0-1**	**54 (90%)**
	2	4 (6.7%)
	>2	2 (3.3%)

We have assessed continence results at months 1, 3 and 6 regarding possible predictors. Incontinent patients (1 or more pads used per day) at 3 and 6 months of follow-up were significantly older at univariate analysis. Table-6 shows these comparisons at 3 months of follow-up. For other variables, there was no statistically significant association.

**Table 6:** Comparison of factors according to continence status 3-months.

Factor		Univariate		
		Continent (n= 39)	Incontinent (n=21)	P
Age		61.93 (5.16)	65.39 (6.57)	0.028
Prostate size		42.48 (18.57)	37.67 (24.56)	0.491
IMC		26.43 (3.70)	26.41 (3.50)	0.991
EBL		104.1 (167.03)	80 (143.49)	0.578
Pathological staging	pT2	34 (87.2%)	14 (66.7%)	0.12
	pT3–pT4	5 (12.8%)	7 (33.3%)	
**NVB preservation**	**Total**	**22 (61.1%)**	**9 (47.4%)**	**0.489**
	**Non-total**	**14 (38.9%)**	**10 (52.6%)**	

**BMI =** body mass index; **EBL =** estimated blood loss; **NVB =** neurovascular bundle

Table-7 shows adjusted odds ratios estimated from a model of univariate logistic regression. The variables with p-value <0.25 were included on the multiple logistic regression model, where older age (p=0.02) and higher pathological staging (p=0.04) were associated to incontinence at 3 months. All the other clinical and pathological characteristics (nerve-sparing status, estimated blood loss, BMI and prostate size) were similar between groups at 3 months. There was no association of factors with continence at first and sixth months post-operative. Nerve sparing technique did not affect continence status in this series of patients.

**Table 7:** Logistic regression analyses of 3-month urinary continence factors.

Factor	Univariate				Multivariate			
	P	OR	95% CI		P	OR	95% CI	
Age	0.03	1.12	1.01	1.25	0.02	1.13	1.02	1.28
Prostate size	0.48	0.98	0.95	1.02			
BMI	0.99	0.99	0.85	1.16			
EBL	0.57	0.99	0.99	1.00			
Pathological staging	0.06	3.4	0.93	13.29	0.04	4.58	1.12	21.67
Nerve sparing status	0.33	1.74	0.56	5.47			

**BMI =** body mass index; **EBL =** estimated blood loss

## DISCUSSION

In the present study we analyzed the perioperative, short and mid-term outcomes and complications related to VUA during learning curve of RARP and compared them with the literature. To our knowledge this is the first study presenting early experience with RARP in a public university hospital in Brazil.

Previous reports showing results from learning curve have suggested an increased rate of complications, and that important outcomes as blood loss and positive margin status would improve after 100 or 250 cases, suggesting a steep learning curve (13). The number of surgical cases needed to achieve a low rate of complications related to the anastomosis is not clearly defined. In a single surgeon series, Ou et al. showed that the learning curve for significantly decreasing overall complications was 150 cases (14).

In Brazil, a few published studies have reported experiences with RARP, and none of them focused on VUA outcomes and complications. The first published paper discussing RARP in Brazil was in 2009 by Colombo Jr and from a private hospital. Results from these series are discussed here.

Several factors influence VUA quality and one of them is the type of suture material. Recently, two meta-analysis comparing barbed sutures (BS) to conventional monofilament sutures for VUA indicated shorter anastomosis time, operative time, and equivalent postoperative leakage rate, estimated blood loss, length of stay, and continence rates (15, 16). The authors deemed that it is easier doing the VUA with BS than with conventional sutures, and concluded that it is an important consideration especially for the novice surgeon (16).

Another factor that may influence continence and complications related to VUA is the type of reconstruction performed. Recent studies have suggested that a more complex posterior and anterior reconstruction of the peri-urethral structures could improve early continence with a low incidence of complications (17, 18). The quality of nerve-sparing has also shown to influence postoperative continence (19).

During their early experience, Artibani et al. reported mean blood loss of 400mL, with 9.8% of the patients receiving blood transfusions (20). In the first reported series in Brazil, Colombo Jr et al. reported mean estimated bleeding of 480mL (100-1800) and transfusion necessary in two patients (2%). One study from Tobias-Machado and another from Lott found mean blood loss of 245.6mL and 212mL respectively (21, 22). In our series, mean EBL was 95mL (±158), no patients had blood transfusions during surgery, but two (3.3%) required it during postoperative period.

Regarding operative times in initial experience series, surgical duration reported in previous studies is extremely variable. One study that evaluated the first 100 cases in a secondary hospital, showed mean VUA time of 47.9 min. and mean console operative times of 225 min. (23). Reports from initial series in some high volume centers showed median surgical duration from 215 to 274 minutes (24, 25). Series from our country reported mean or median surgical times from 175 to 298 minutes (3, 21, 22, 26). In 2005 Patel et al. described an extremely short mean operative time of 141 min. in their initial 200 cases (27). In the present series, mean total console time, and time to perform anastomosis, were 298 and 34 minutes respectively.

Our study presents results from our initial experience with RARP in a laparoscopic naive center. During these procedures, lessening surgical time was not a main goal compared to achieving good functional and oncological results. This reflected on our times of whole surgery and time to complete anastomosis being longer than current series of experienced surgeons, but allowed a very low incidence of complications related to the anastomosis, with no bladder neck sclerosis, no retention, and only 2 urinary leakage that did not require any intervention for its treatment. Another factor influencing total operative time is that most patients (65%) required pelvic lymph node dissection. We usually perform standard or extended PLND on patients with intermediate or high risk of progression, respectively.

Regarding these mid-term complications our results were also comparable to current literature. In 2008, Artibani and colleagues published their initial 41 cases and described one bladder-urethra anastomosis dehiscence, which required reintervention, and one bladder-urethra anastomosis stenosis using monofilament suture (20). Recently Jacobsen et al. reported their data from 236 consecutive patients, with an anastomotic leakage frequency of 2.9%, and anastomotic stricture of 4.9% (28). In another study that assessed complications during early experience of RARP, among 322 patients, urine leakage developed in 24 (7.5%) and anastomotic strictures requiring transurethral incision developed in 2 cases (0.3%) (29). One study that evaluated first 100 cases performed by 5 surgeons in a private hospital in Brazil, reported zero cases of leakage or bladder neck contraction (3).

Together with a low incidence of complications and apart from longer operative times than other series, our continence results were comparable to current literature. Publications from high-volume centers have found rates of continence (up to 1 pad/day) at 6 months of 71.7% to 93% on initial series (24, 30). One report from Brazil found 6 months continence rates of 93.3% (21). Lott et al. described continence rates of 88% in 6 months (22). Considering the criterion of continence up to 1 security pad, the present series showed 90% continence at 6 months.

Regarding factors predictive of return to continence, we found that patient age and pathological staging were significantly associated with 3-month continence status on multivariable analysis.

Patient age and Charlson comorbidity index have been found to be significantly associated with 12-month continence status after RARP in previous studies (31). Higher quality nerve-sparing was found to be associated positively with 1 year continence and to patients’ perception of urinary recovery as measured by EPIC urinary outcome scores (19, 32).

Possible reasons for not finding a statistically significant association between the degree of preservation of neurovascular bundles and urinary incontinence are our sample size, lack of standardized score for the degree of preservation and lack of data on 12 months continence. Other factors that might affect continence outcomes were not included in our study such as bladder neck preservation, membranous urethral length, thickness of the *levator ani* muscle and urogenital diaphragm, higher pre-operative IPSS, type of urinary sphincter complex reconstruction, comorbidity score, among others, so that is a possible limitation of the model. Also, we did not use validated questionnaires for continence evaluation, although the number of pads/day is a broadly used variable, and the result of 78.3% of patients using no pads at 6 months represents a consistent finding considering an initial experience series.

Apart from these challenges and limitations, this was a prospective study with strict follow-up. Our main results are comparable to the literature, and reflect the experience and results starting from the very first case performed in a public training hospital. We believe that these results are relevant for other centers initiating their urologic robotic program. Our next goal is to compare these outcomes and other variables together with our open radical prostatectomy cases, and these data are being gathered for next publications.

## CONCLUSIONS

Our data indicate that, during early experience with RARP in a public academic hospital, it is possible to achieve good continence results with low rates of bleeding and complications related to vesicourethral anastomosis. We also found that age and pathological staging was associated to early continence status.

## ABREVIATIONS

RP: = radical prostatectomy
RARP: = robotic assisted radical prostatectomy
VUA: = vesicourethral anastomosis
PLND: = pelvic lymph node dissection
RS: = Rocco stitch
EBL: = estimated blood loss
TRUS: = transrectal ultrasound
PSA: = prostatic specific antigen
BS: = barbed sutures
